# Evidence That Emotion Mediates Social Attention in Rhesus Macaques

**DOI:** 10.1371/journal.pone.0044387

**Published:** 2012-08-30

**Authors:** Emily J. Bethell, Amanda Holmes, Ann MacLarnon, Stuart Semple

**Affiliations:** 1 Centre for Research in Evolutionary and Environmental Anthropology, University of Roehampton, London, United Kingdom; 2 Department of Psychology, University of Roehampton, London, United Kingdom; Indiana University, United States of America

## Abstract

**Background:**

Recent work on non-human primates indicates that the allocation of social attention is mediated by characteristics of the attending animal, such as social status and genotype, as well as by the value of the target to which attention is directed. Studies of humans indicate that an individual’s emotion state also plays a crucial role in mediating their social attention; for example, individuals look for longer towards aggressive faces when they are feeling more anxious, and this bias leads to increased negative arousal and distraction from other ongoing tasks. To our knowledge, no studies have tested for an effect of emotion state on allocation of social attention in any non-human species.

**Methodology:**

We presented captive adult male rhesus macaques with pairs of adult male conspecific face images - one with an aggressive expression, one with a neutral expression - and recorded gaze towards these images. Each animal was tested twice, once during a putatively stressful condition (i.e. following a veterinary health check), and once during a neutral (or potentially positive) condition (i.e. a period of environmental enrichment). Initial analyses revealed that behavioural indicators of anxiety and stress were significantly higher after the health check than during enrichment, indicating that the former caused a negative shift in emotional state.

**Principle Findings:**

The macaques showed initial vigilance for aggressive faces across both conditions, but subsequent responses differed between conditions. Following the health check, initial vigilance was followed by rapid and sustained avoidance of aggressive faces. By contrast, during the period of enrichment, the macaques showed sustained attention towards the same aggressive faces.

**Conclusions/Significance:**

These data provide, to our knowledge, the first evidence that shifts in emotion state mediate social attention towards and away from facial cues of emotion in a non-human animal. This work provides novel insights into the evolution of emotion-attention interactions in humans, and mechanisms of social behaviour in non-human primates, and may have important implications for understanding animal psychological wellbeing.

## Introduction

People’s emotion state strongly influences their allocation of social attention [1], [2], and this plays a fundamental role in shaping their social interactions [3]–[5]. Moreover, specific patterns of bias in attention are associated with clinical models of psychological wellbeing and associated pathologies [1]–[3], [5]–[8]. For example, humans have a bias to attend preferentially to signals of threat, such as angry faces compared with neutral faces [1], [9]. This attentional bias for threatening stimuli may provide a fitness benefit in terms of faster detection of threat and therefore improved ability to defend against, or escape, danger [10]. Experimental evidence has shown that attentional bias for threat is further enhanced in individuals with increased levels of anxiety: people who report higher levels of state anxiety look for longer towards aggressive faces compared with neutral distractors [2] and are faster to detect a probe that appears at the location of an aggressive face than they are to detect the same probe at the location of a neutral face [6]. Enhanced vigilance for threat while in an anxious state has been proposed as a mechanism for adaptive modulation of behaviour according to the degree that the surrounding environment is perceived as threatening [3], [10]: it is adaptive to become more fearful or anxious in a dangerous environment and consequently to be more vigilant for signals of threat. By contrast, in a safer environment, fearfulness and anxiety are reduced and attentional resources are directed towards other fitness-relevant stimuli (e.g. food or mating opportunities [11]).

While enhanced vigilance for threat with increased state anxiety is considered an adaptive response to acute stressors, it has also been implicated in the onset and maintenance of anxiety disorders in humans [1], [3], [8], [12], [13]; increased vigilance for threat results in an elevated perception of threat [12] which leads to further increases in anxiety [13]. Over time, chronically elevated levels of anxiety and vigilance towards threat may reach a threshold beyond which the individual is unable to cope with any further increases in anxiety, culminating in the strategic avoidance of anxiety-eliciting stimuli [3], [14]. Avoidance of threat cues is characteristic of clinical conditions, such as social phobia and social isolation, and maintains such conditions through reduced opportunity for desensitization to the fear-inducing stimuli [2], [3], [8], [14]. The way in which emotion mediates social attention in humans is therefore central to human psychological wellbeing.

Despite the known importance of emotion-mediated attentional biases in humans [1]–[9], and speculation about the importance of their role in the evolution of human social behaviour [3], [10], [15], whether short-term shifts in emotion mediate social attention in any other species of animal has not yet been tested. Recently, two converging lines of research have called for the introduction of the kind of attention-orienting approaches typically used in clinical studies of humans: to support the development of new animal models of human psychopathology [16] and to provide novel measures of non-human animal psychological wellbeing in its own right [17], [18]. There are arguments that such approaches may help elucidate the attentional and cognitive deficits underlying human psychopathologies and emotional disorders such as schizophrenia, depression, anxiety and autism [16]. At the same time, they may also help to clarify components underlying psychological wellbeing for the research species themselves, informing the reduction and refinement in the use of animals in research [18]. Given the widespread use of non-human primate models of human social attention and associated disorders [10], [16], [19]–[27], it is crucial to understand the similarities and differences between human and other primates in how social attention is deployed [28], [29]. Experimental studies of non-human primate social attention have revealed that allocation of social attention is mediated by characteristics of the attending animal, such as social status [24], genotype [19], [26] and recent social experience [30], as well as by the value of the target to which attention is directed [20]. However, no study, to our knowledge, has applied these methods to test explicitly the effect of short term changes in emotion on social attention in a non-human primate.

We tested for evidence that a shift in emotion state - specifically an increase in anxiety/stress resulting from a veterinary health check involving physical restraint and injection with ketamine hydrochloride - leads to changes in social attention in captive adult male rhesus macaques. We predicted that, when shown pairs of conspecific faces (one with an aggressive and one with a neutral expression), monkeys would show a general vigilance for the aggressive face, but that maintenance of attention (continued vigilance or switch to avoidance) would vary according to whether the viewing monkey had recently undergone the (putatively negative) health check. Initially, we recorded behavioural indicators of anxiety and stress after the health check and during a period of (putatively neutral or positive) standard husbandry including environmental enrichment; we compared these behavioural measures to test our prediction that the health check would cause a negative shift in emotion state. Then we measured and compared eye-gaze patterns of the macaques as they viewed aggressive-neutral face pairs after the health check, and during the period of enrichment.

## Results

### Behavioural Indicators of Negative Emotion State Increase after the Health Check

**Figure 1:**
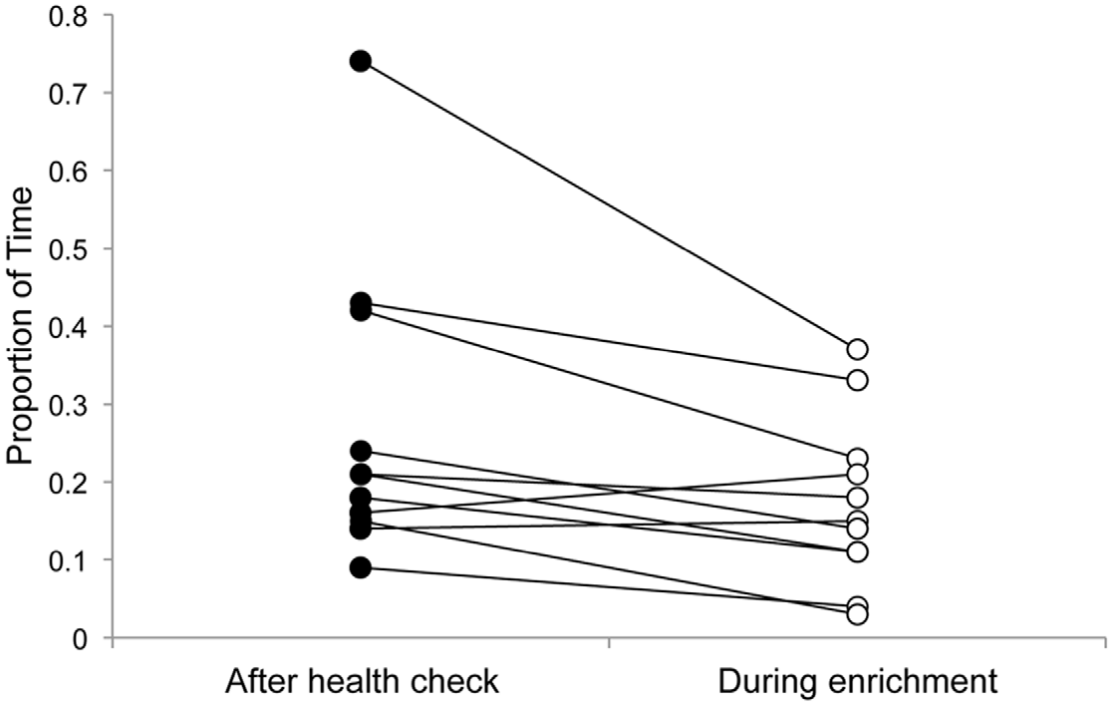
Behavioural indicators of emotion state. Proportion of time monkeys (n = 11) engaged in self-directed, stereotypical and self-injurious behaviours after the health check and during the period of enrichment. Lines join the two data points for each animal.

Monkeys spent a significantly greater proportion of time engaged in behavioural indicators of anxiety and stress (self-directed, stereotypical and self-injurious behaviours) on the day of attention testing and consecutive two days following the health check compared to the day of attention testing and subsequent two days during the period of enrichment (Z = 2.401, *p* = 0.016, Figure 1). This supports our prediction, and findings from previous studies (see Methods), that the procedures involved in the health check led to a negative shift in emotion state, and that this shift lasted beyond the duration of the experimental testing sessions.

### Direction of First Gaze does not Differ between Conditions

Binary GLMM revealed monkeys’ tendency to direct initial gaze to the left or right stimulus of the aggressive-neutral face pair was not influenced by aggressive face location or condition (all *p *values >0.09), indicating that neither the emotion content of the faces, visual field in which stimuli were presented, nor the emotion state of the viewing monkey affected direction of first gaze.

### Latency to First Gaze is Faster towards Aggressive than Neutral Faces

**Figure 2:**
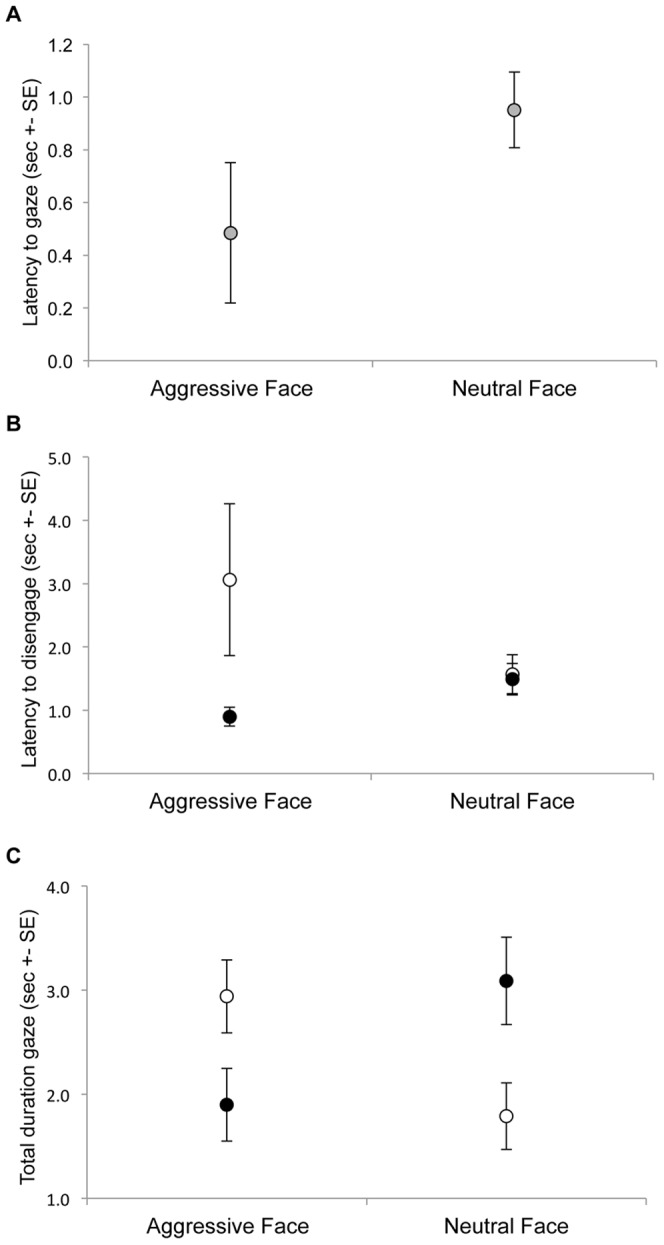
Social attention for aggressive-neutral face pairs. (A) Latency to gaze towards the aggressive or neutral face on experimental trials when each was the first stimulus to be looked at (pooled across conditions). (B) Latency to disengage first gaze from aggressive and neutral faces on experimental trials after the health check (filled circles) compared with during the enrichment condition (open circles). (C) Total duration of gaze towards aggressive and neutral faces after the health check (filled circles) and during the enrichment condition (open circles). All data indicate mean seconds± s.e.m.

Monkeys were significantly faster to direct initial gaze towards aggressive than towards neutral faces when these were first to be looked towards (0.49s ± 0.27 and 0.95s ± 0.14, respectively; permutation test: n = 7; *p* = 0.03: Figure 2A), indicating a rapid vigilance for aggressive faces. This rapid vigilance was apparent during the period of enrichment (permutation test, n = 7; *p* = 0.05) but not following the health-check (permutation test, n = 5; *p* = 0.30).

### Latency to Disengage First Gaze away from Aggressive Faces is Faster following the Health Check

GLMM performed on the mean latency to disengage gaze from aggressive and neutral faces revealed a significant main effect of testing condition (*F*_1,54_ = 7.07, *p* = 0.01) and a near-significant interaction of face x condition (*F*_1,54_ = 3.41, *p* = 0.07: Figure 2B). Simple contrasts revealed that monkeys were significantly faster to disengage first gaze from aggressive faces after the health check than during the period of enrichment (0.90s ± 0.15 and 3.06s ± 1.20, respectively; permutation test: n = 7, *p* = 0.001). There was no difference in latency to disengage first gaze from neutral faces between the two conditions (health check: 1.49s ± 0.25; enrichment: 1.57s ± 0.31; Permutation test: n = 7, *p* = 0.88). After the health check, there was a trend to disengage first gaze faster from aggressive than neutral faces (0.90s ± 0.15 and 1.49s ± 0.25, respectively; permutation test: n = 7, *p* = 0.06). There was no difference in latency to disengage first gaze from aggressive versus neutral faces during the period of enrichment (3.06s ± 1.20 and 1.57s ± 0.31, respectively; permutation test: n = 7, *p* = 0.31). These data suggest faster disengagement from aggressive faces following the health check.

### Total Duration of Gaze towards Aggressive Faces is Lower following the Health Check

GLMM performed on the mean duration of gaze towards aggressive and neutral faces revealed a significant interaction of face x condition (*F*_1,56_ = 10.87, *p* = 0.002: Figure 2C), with no significant main effects. Simple contrasts revealed that monkeys spent less time looking towards aggressive faces after the health check than they did in the enrichment condition (1.90s ± 0.35 and 2.94s ± 0.35, respectively; permutation test: n = 7, *p* = 0.04). Conversely, monkeys spent more time looking towards neutral faces after the health check than during the period of enrichment (3.09s ± 0.42 and 1.79s ± 0.32, respectively; permutation test: n = 7, *p* = 0.02). After the health check, monkeys spent significantly less time gazing towards aggressive than neutral faces (1.90s ± 0.35 and 3.09s ± 0.42, respectively; permutation test: n = 7, *p* = 0.03). Conversely, during the period of enrichment duration of gaze was longer towards aggressive versus neutral faces (2.94s ± 0.35 and 1.79s ± 0.32, respectively; permutation test: n = 7, *p* = 0.04). These data suggest overall avoidance of aggressive faces after the health check and overall vigilance for aggressive faces during the enrichment period.

## Discussion

Our results provide, to our knowledge, the first systematic evidence that changes in emotion state mediate social attention for facial expressions of emotion in a non-human animal: the way in which rhesus macaques visually attended to conspecific faces varied as a function of both the viewer’s inferred emotion state and the emotion content of the faces. These findings have implications for extending our understanding of macaque cognition and behaviour, and the nature and evolution of human attentional bias. They may also have important potential applications for our understanding of primate models of human attention-related affective disorders, and for the assessment of captive primate welfare.

The monkeys in our study were faster to direct initial gaze towards aggressive than neutral faces; an initial orienting bias apparently driven by the enriched condition, but not the health-check. It is possible that, following the health-check, attentional avoidance was instigated even before fixation of gaze on the aggressive stimulus. The overall vigilance for aggressive faces suggests that the macaque brain possesses systems dedicated to the preferential processing of facial expressions of emotion. This is a bias that, although suggested [15], [31], has never previously been demonstrated using the kind of paradigms that have been widely used with humans. Our finding is in line with neurophysiological [9], reaction time [1] and eye-gaze [1], [2] data from humans indicating rapid vigilance for emotional versus neutral faces. This supports evidence from human and non-human animals for an evolved threat-detection system which functions automatically and independently of emotion state, especially at early stages of processing [8]–[11], [15]. Extending the current study to include non-social threatening (e.g. predator) stimuli would allow us to test whether the patterns of attention revealed here reflect a response to social threat specifically, or a more generalized threat response.

Although there was a bias in the speed of initial gaze towards aggressive faces, monkeys were no more likely to orient first gaze towards aggressive faces. The lack of evidence for this latter bias may be due to the stimuli being presented with a lateral separation that may have hindered initial capture of attention. Studies revealing a bias in orienting towards aggressive versus neutral faces in humans present both stimuli within the central/parafoveal fields of view (interstimulus distances between center points of stimuli typically range between 100 mm and 186 mm [2], [6]). In the current study, stimuli were presented on two screens with an interstimulus distance of 450 mm. This allowed reliable discrimination of gaze direction during video coding, but meant stimuli were presented peripherally, outside the central/parafoveal fields of view. At peripheral locations, stimulus processing is degraded in both humans [32] and macaques [33]). We suggest further studies sensitive to covert orienting towards stimuli presented at shorter inter-stimulus distances (i.e. on a single screen), are required to examine these initial orienting effects in more detail. Further, the development of related paradigms such as Stroop-like interference tasks [1], [34] will allow exploration of different aspects of attention (viz. attentional capture versus spatial attention).

Following initial orientation towards aggressive faces, monkeys that had recently undergone the health check more rapidly disengaged gaze from aggressive face stimuli and spent less time looking towards aggressive faces overall, compared to when the same animals were tested during a phase of standard environmental enrichment. Importantly, following the health check monkeys showed a near-significant trend to be faster to disengage first gaze, and spent less time looking towards aggressive faces, compared with neutral faces, suggesting avoidance of presumably threatening social information relative to more neutral social stimuli. Gaze aversion is an important signal of submission in macaques [35] and previous work suggests the tendency to avert social gaze in macaques has genetic [26] and developmental [35] correlates, which may interact with one another [19]. Our results provide novel evidence that short-term changes in emotion state following an environmental stressor (restraint; as evidenced by an increase in stress-related behaviours) may also influence gaze towards social stimuli. We propose this altered attention towards (or away from) social stimuli is a mediating link between emotion state and behavioural response, that may drive behavioural flexibility in social interactions as seemingly complex as reconciliation and cooperation [30], [36], [37]. For example, emerging data from humans suggest that, under experimental conditions, competition-dependent acute changes in testosterone levels in ‘winners’ and ‘losers’ [38], [39] may also be accompanied by shifts in selective attention for threatening faces [40]. Male rhesus macaques face high levels of competition for access to resources such as sexually receptive females, and degree of competition for access to mates predicts variation in male testosterone levels [41]. It may be that defeat in contests with concomitant changes in testosterone-related selective attention for emotional faces would cause male rhesus macaques to avoid engaging in future dominance interactions. A win may result in a testosterone-related enhanced selective attention and approach towards threat (e.g. [34], [38], [42], [43]), possibly with some modulating effects of social status [38] and genetic profile [39]. However, evidence for a causal relationship between testosterone and social attention for face cues to threat in non-human primates is currently lacking [42], and we are only just beginning to understand the genetic and other physiological correlates of social attention in humans and some other species [19], [26], [27], [34], [39], [44]–[46].

Recent models of human attentional processes have emphasized a role for initial stimulus evaluation processes in directing attention to social stimuli, with an emphasis on how anxiety will cause mildly threatening stimuli to appear even more threatening [1], [8], [12]. It may therefore be the case that the shifts in emotion state following the experimental manipulations used here were accompanied by concomitant changes in the emotion evaluation (i.e. relevance) of the faces. For example, a heightened sensitivity to perceived threat following the health check may account for the pattern of avoidance of aggressive faces. According to this line of reasoning, and in line with cognitive models of emotion-cognition interaction [1]–[6], early shifts in attention may be driven by early low-level stimulus appraisal processes with model-specific predicted outcomes in terms of orienting of attention towards or away from threat. Most theories suggest threatening stimuli capture attention in all individuals [10], and some cognitive models predict specific appraisal outcomes may depend in part on characteristics of the individual, such as anxiety state [1]–[6]. In addition, the relevance of an aggressive face (social threat) may be equivalent (and immediate) to all monkeys following restraint, but the relevance of an aggressive face during a phase of environmental enrichment may be subject to mediation by additional factors such as individual differences in motivation, temperament and dominance [24], [26]. This may explain the large degree of variation in latency to disengage gaze from aggressive faces during the period of enrichment. We are currently exploring possible trait factors underlying this variation.

Our results are also relevant to recent attempts to develop a picture of the cognitive endophenotype of human ancestors [47]. Human comparative studies have suggested that variation in allelic frequency of genes linked to emotion-mediated biases in attention and cognition [45] may have co-evolved with cultural differences between human populations [47]. The present data push back the link between emotion and social attention to an earlier point on the evolutionary tree than has previously been demonstrated. Our findings illustrate an important role for data from extant species of non-human primates in developing our understanding of the emergence of emotional, attentional and cognitive traits linked to human cultural variation.

In humans, particular patterns of attentional bias for social information are associated with psychopathology [1], [3], [5], [8] and macaques are a widely used research model in this area [16]. People suffering from clinical levels of social anxiety show an initial vigilance followed by a rapid and overall avoidance of threatening (versus neutral) faces [2]. This ‘vigilance-avoidance’ [1]–[3] is implicated in the onset and maintenance of anxiety disorders: initial vigilance for threat results in a high rate of threat detection, while subsequent avoidance may impair habituation to fear-relevant stimuli and lead to elevated anxiety, accumulating over time to produce clinical levels of social anxiety [3] and paranoid delusions in schizophrenia [8]. This maladaptive response ultimately impairs quality of social interactions and can have a profound impact on quality of life [2], [3], [5]–[8]. The finding in the present study of patterns of emotion-mediated avoidance of threatening faces in a non-human primate will be of interest to those using animal models of a range of widespread and debilitating human psychological disorders.

The use of a repeated measures design in our study raises an important point regarding methodology. Studies with humans largely use a between-subjects design; the expression of attentional bias is commonly investigated among individuals who score high or low in state or trait affect, as measured using questionnaires [1], [2], [5], [6]. Some evidence from humans suggests that experimentally induced shifts in attentional biases result in shifts in state affect [13], [48], and increased vulnerability to anxiety following real-life stressors [48]. However, no studies have investigated, as we have here with non-human primates, whether *a priori* shifts in emotion state within subjects may lead to the type of shifts in attentional bias that have been linked to the onset of human psychopathology. Our data suggest this is worth investigating in humans.

The current findings also have important implications for our understanding and management of the psychological wellbeing of animals in captivity [17]. During the present study, monkeys showed an avoidance of threatening faces on the day after a health-check. If husbandry procedures such as routine health-checks impair monkeys’ subsequent abilities to attend appropriately to social interactants, this presents an important consideration for the way in which animals are managed. Adaptation of the present method using stimuli associated with the captive environment and husbandry procedures could elucidate which factors capture attention and may therefore act as the greatest stressors to captive animals. Furthermore, we predict that future development of this method, incorporating human attention bias modification paradigms [13], [48] for use with non-human animals, will open the door to a range of therapeutic, as well as diagnostic, tools for improving animal welfare.

Our results call for further investigation of emotion-mediated attentional biases for social information across non-human animals, and exploration of the underlying mechanisms. A natural extension of the current study would be its application under more species-typical environmental conditions. Studies of free-ranging male rhesus macaques’ responses to conspecific face pairs [49], [50] indicates that such an approach is indeed feasible, and could be carried out without any need for training of the animals involved. In addition video playback could be used to explore attentional bias to dynamic social situations [51], [52]. Finally, this work highlights the need for future studies of social attention to consider how emotion may interact with intrinsic and extrinsic factors, such as genotype [19], [26], [45], social status [24], [53], hormone levels [34], [42], [46], previous social experience [2], [30] and the value of the social target [20], [23] to which attention is directed.

## Materials and Methods

### Ethics Statement

All work was conducted in accordance with the recommendations of the Weatherall Report ‘The use of nonhuman primates in research’ and under approval of the University of Puerto Rico Medical Sciences Campus IACUC (A1850106) and the ethics committee of the University of Roehampton. Since developing a novel measure of animal welfare was the primary goal of the research project of which this study was a part, care was taken to use positive reinforcement methods that are considered to provide the best approach from an animal welfare perspective [54]. Only animals that voluntarily entered the testing cage for food rewards took part in the study and at no point were animals negatively reinforced or coerced into taking part in the study. The study was timed to take place around pre-existing veterinary health-checks which meant there was no need to introduce a ‘negative’ emotional manipulation solely for purposes of this study. We did introduce environmental enrichment, widely used to improve welfare in captive macaques [54], to induce a relative ‘positive’ shift in emotion state.

### Animals, Housing and Training Procedure

Study animals were initially eight singly-housed adult male rhesus macaques (range: 5–23 years old; average age: 10±6 years), housed at the Sabana Seca Field Station, Caribbean Primate Research Center, Puerto Rico. All animals were naïve to cognitive testing procedures at the start of training. Initially, each monkey was trained, using food-based positive reinforcement, to enter a testing cage for transportation to a laboratory where he was positioned in front of two screens. Pairs of images of female rumps were presented on the screens to encourage attention towards the apparatus, after which the monkey was left undisturbed to feed on the daily food ration. Monkeys who did not initially attend to rump images presented on the screens were encouraged to do so by the occasional delivery of a primate pellet via a chute from a concealed automated pellet dispenser into a well, situated centrally between the two screens. Only monkeys who learned to enter the cage for a small food reward and fed on the daily food ration while in the laboratory took part in the study (n  = 7).

### Stimuli and Apparatus

Face stimuli were compiled from 20 colour photographs of 10 adult male conspecifics who were unfamiliar to the study animals. For each stimulus monkey, one photograph showed a frontal view of the face with aggressive expression, and one photograph showed a frontal view of the face with neutral expression. Face pictures were cropped around the face and matched for size before being superimposed on a grey background and enclosed in a rectangular frame measuring 154×164 mm when presented on a 16 in. computer monitor. Aggressive faces did not differ from neutral faces in either luminosity (t_9_ = 0.97, *P*  = 0.36) or contrast energy (t_9_ = 2.20, *P*  = 0.92). The face stimuli were paired, according to stimulus monkey identity, to give 10 aggressive-neutral face pairs. From each of the 10 neutral face stimuli a scrambled face stimulus was compiled by decomposing and randomly reassembling each face such that the configuration of facial features was disrupted but the surface properties (luminosity and contrast energy) remained the same.

**Figure 3:**
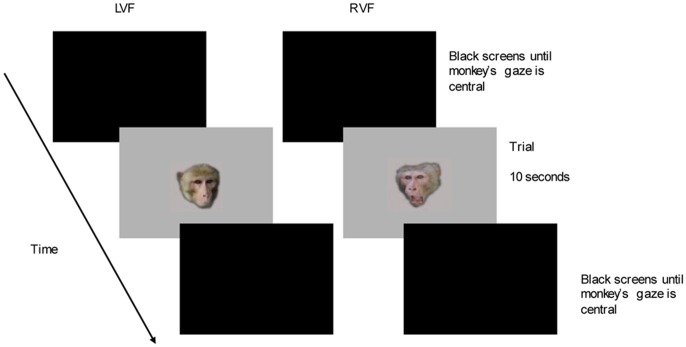
Example of an experimental trial showing an aggressive-neutral face pair.

Neutral-aggressive face pairs were presented on two adjacent Sony 16in. computer monitors (one face per monitor: Figure 3). The computer monitors were positioned to the left and right of a pellet tray which was connected via a chute to a concealed pellet dispenser**.** The horizontal distance between the mid-points of the two screens was 45 cm, so that the distance of each stimulus’ mid-point from the central line of fixation was 22.5 cm. The illuminance readings did not differ between the two screens (paired samples t-test: t_29_ = 0.15, *P*  = 0.88). The two computer monitors were connected via a junction box with split-screen monitor to a Satellite Pro A60 laptop. The junction box, split-screen monitor and laptop were all situated in an adjacent control room, from where the experimenter ran the experimental session. All sessions were filmed using a Samsung VP-L150 digital video camera placed centrally behind the two monitors. The digital video camera was positioned to film the monkey’s direction of gaze, and a live video feed to the control room allowed the experimenter to observe the monkey on a video monitor. Two small mirrors on the front of the cage allowed stimulus onset and offset to be detected on the video, for the purposes of later blind-coding. The consistent alignment between the camera and two screens (the inner top corners of which were visible on the lower corners of the video), and position of the monkey centrally between the screens, enhanced coding efficiency; focused gaze and social responses to stimuli were used during the initial calibration to ascertain when monkeys were looking at stimuli presented on either screen.

### Experimental Design and Procedure

A daily testing session comprised 21 trials (experimental and control) presented in a randomised order. There were six experimental trials (aggressive-neutral face pairs) in a daily testing session, counterbalanced for equal presentation of the aggressive face to the left visual field (left hand monitor) and the right visual field (right hand monitor). Additional control trials on which the same image was presented on both screens (3 x aggressive-aggressive, 3 x neutral-neutral, 3 x scrambled-scrambled) were included to prevent habituation to the aggressive-neutral experimental trials and to test overall differences in patterns of gaze to emotional compared with neutral stimuli. An additional six control trials on which neutral-scrambled face pairs were shown were also included to investigate attention for social versus non-social information. Analyses of control trials showed no significant effects and are therefore not considered further.

The procedure for a testing session was as follows. The monkey was transported to the laboratory in the testing cage, positioned in front of the apparatus and allowed to settle. The experimenter immediately moved to the adjacent room and set the video to record events. The monkey was encouraged to gaze centrally, between the two screens, by the delivery of a single primate pellet into the pellet tray. Once the monkey gazed centrally between the screens the experimenter triggered the onset of the first trial. On each trial, the stimulus pair was presented for 10 seconds and the monkey’s gaze towards the stimuli filmed. Following stimulus offset, a black screen appeared simultaneously on both monitors. The next trial began when the monkey next looked centrally between the screens, which they did frequently by chance. Subsequent pellets were only delivered into the pellet tray on the rare occasions when monkeys failed for several minutes to look centrally.

### Emotion Manipulations and Behavioural Measures

Monkeys were tested during each of two conditions: once on the day following restraint for a health check (a routine veterinary practice involving procedures shown to induce behaviours associated with anxiety in rhesus macaques [55]) and on the eighth day of a 10 day period of standard husbandry which included provision of environmental enrichment (which has been shown to induce changes in behaviour associated with reduced anxiety and improved welfare in rhesus macaques [54]). During the health check, each monkey was restrained in the home cage by the veterinarian, injected for anaesthesia with Ketamine Hydrochloride (KHCl) then removed for examination. During the enrichment condition, monkeys were provisioned with the daily food ration in feeding devices designed to increase opportunity for manipulation of food and exploratory behaviour. Order of testing was counterbalanced across these two conditions so that four monkeys were tested after the health check first and three during the enrichment period first.

The prediction that the health check would lead to a negative shift in emotion state was tested using behavioural measures. In particular, data were collected on time spent engaged in self-directed, stereotypical and self-injurious behaviours; these behaviours have been widely associated with anxiety and stress in a range of observational, physiological, and pharmacological studies in rhesus macaques [56]–[61] and other primate species [62]–[64]. A total of 11 monkeys, five of whom took part in the experimental sessions, were observed in the home cage using focal animal continuous sampling [65]. Each monkey was observed during one morning and one afternoon five minute observation session for each of three days immediately following the day on which they underwent the health check (i.e. the day of testing and the subsequent two days). Data were also collected from the day of testing and the subsequent two days during the enrichment period (i.e. days 8–10 of enrichment). Behavioural data were recorded on an IBM ThinkPad 755CD notebook computer using JWatcher™ 0.9 software.

### Video Coding

Video from experimental sessions was blind coded by two observers on a frame by frame basis using iMovie HD version 6.0.3 software on an AppleMac computer. Video was coded for: direction of first gaze (either towards the stimulus on the left screen or stimulus on the right screen); latency to direct the first gaze towards a stimulus (calculated as the time to shift gaze from the central location at stimulus onset towards one of the two stimuli); latency to disengage first gaze (calculated as the time to look away following both first gaze to the left screen stimulus and also first gaze to the right screen stimulus); total duration of gaze towards both the left and also the right screen stimulus (calculated as the sum of all gaze bouts made within each 10 second trial). Total duration of gaze and latency to disengage first gaze are two measures of the ‘overt’ maintenance of visual attention (i.e. involving eye movement in the direction of the attentional shift) while direction and latency of first gaze are two measures of ‘overt’ initial allocation of visual attention. Inter-observer reliability for direction of gaze towards the two screens was attained at Cohen’s *k* = 0.76. Once coding was completed, trials were identified as experimental (aggressive-neutral face pairs) or control (e.g. neutral-neutral) and, for experimental trials, whether the aggressive face was presented on the left or right screen.

### Data Analyses

First, to explore changes in emotion state related to the health check, for each monkey, mean percentage of time spent engaged in self-directed, stereotypical and self-injurious behaviours was calculated across the three days after the health check and across the three days during the enrichment condition. Data were pooled across the three days in each condition due to the low frequency with which these behaviours occurred. Data were compared between the two testing conditions using a Wilcoxon matched pairs signed rank test.

Data from experimental trials (aggressive-neutral face pairs) were analyzed using a Generalized Linear Mixed Model (GLMM) [66], [67] with testing condition (after health check, during the period of enrichment), face (aggressive, neutral) and aggressive face location (left visual field, right visual field) as fixed repeated measures factors, and animal ID as a random covariate (Table 1). Simple contrasts were performed to explore significant interaction effects using a permutation test for related samples [68]. The test is a variant of Friedman’s one-way ANOVA, except that empty cells are retained in a series of 10,000 permutations. It is designed specifically for data sets with a small sample size and empty cells.

**Table 1:** Variables used in the GLMM analyses.

Variable	Description	Type
**Dependent variables**		
Direction of first gaze	The stimulus to which monkeys first orientedgaze post-stimulus onset	Dichotomous (0 = neutral, 1 = aggressive)
Total duration of gaze	Sum of all looking bouts toward each stimulus per trial	Continuous
Latency to disengage first gaze	Duration of the first looking bout towards each stimulus per trial	Continuous
**Fixed explanatory variables**		
Emotion state manipulation		
- Testing Condition	Testing session was held either during a phaseof enrichment or following restraint for a veterinary inspection	Dichotomous (0 = enriched, 1 = health-check)
Stimulus characteristics		
- Face	Stimuli were presented as aggressive - neutral face pairs	Dichotomous (0 = neutral, 1 = aggressive)
- Aggressive Face Location	Each stimulus was presented an equal number of timesin the left and right visual fields	Dichotomous (0 = LVF, 1 = RVF)
**Random variable**		
Monkey Identity	Seven monkeys took part	Nominal

For direction of first gaze, a GLMM was performed (with the factors described above, but excluding ‘face’) with direction of gaze (left, right) as the binary dependent variable. Only trials in which monkeys were looking centrally between the two monitors at stimuli onset were included in the analyses.

For latency to the first gaze following stimuli onset, mean latency to look towards either stimulus was calculated for each monkey, separately for aggressive and neutral faces. Three monkeys avoided looking first towards the aggressive face in any single trial. This non-random distribution of empty cells constituted a non-ignorable factor [69] which precluded statistical comparisons (GLMM) across conditions using the full monkey cohort. Data were therefore collapsed across the two conditions and a permutation test to compare latency between first gaze towards aggressive and first gaze towards neutral faces was conducted.

Mean latencies to disengage initial gaze from aggressive and neutral faces were calculated and entered into a GLMM. Analyses were performed on first look towards both the aggressive and the neutral face for each trial, regardless of the direction of the very first shift of gaze. Follow-up comparisons using permutation tests were again performed. Finally, for total duration of gaze towards aggressive and neutral faces, a mean was calculated for each monkey and trial type and entered into a GLMM. Simple contrasts were then performed to explore further any significant interaction effects.
